# Cancer‐related knowledge, beliefs, and behaviors among Hispanic/Latino residents of Indiana

**DOI:** 10.1002/cam4.5466

**Published:** 2023-01-22

**Authors:** Manuel R. Espinoza‐Gutarra, Susan M. Rawl, Gerardo Maupome, Heather A. O'Leary, Robin E. Valenzuela, Caeli Malloy, Lilian Golzarri‐Arroyo, Erik Parker, Laura Haunert, David A. Haggstrom

**Affiliations:** ^1^ Division of Hematology and Oncology, Department of Medicine University of Alabama at Birmingham Birmingham Alabama USA; ^2^ Indiana University Melvin and Bren Simon Comprehensive Cancer Center Indianapolis Indiana USA; ^3^ Indiana University School of Nursing Indianapolis Indiana USA; ^4^ Indiana University Purdue University Indianapolis, Richard M. Fairbanks School of Public Health Indianapolis Indiana USA; ^5^ Northeast Ohio Medical University Rootstown Ohio USA; ^6^ Indiana University Purdue University Fort Wayne Fort Wayne Indiana USA; ^7^ School of Public Health Indiana University Bloomington Bloomington Indiana USA; ^8^ Indiana University School of Medicine Indianapolis Indiana USA; ^9^ Center for Health Services Research Regenstrief Institute Indianapolis Indiana USA; ^10^ VA HSR&D Center for Health Information and Communication Richard L. Roudebush Veterans Affairs Medical Center Indianapolis Indiana USA; ^11^ Division of General Internal Medicine and Geriatrics Indiana University School of Medicine Indianapolis Indiana USA

**Keywords:** behaviors, Hispanic, knowledge, screening, survey

## Abstract

**Background:**

Cancer is the leading cause of death for Hispanics in the USA. Screening and prevention reduce cancer morbidity and mortality.

**Methods:**

This study administered a cross‐sectional web‐based survey to self‐identified Hispanic residents in the state of Indiana to assess their cancer‐related knowledge, beliefs, and behaviors, as well as to identify what factors might be associated with cancer screening and prevention. Chi‐square and Fisher's exact test were used to compare associations and logistic regression used to develop both univariate and multivariate regression models.

**Results:**

A total of 1520 surveys were completed, median age of respondents was 53, 52% identified as men, 50.9% completed the survey in Spanish, and 60.4% identified the USA as their country of birth. Most were not able to accurately identify ages to begin screening for breast, colorectal, or lung cancer, and there were significant differences in cancer knowledge by education level. US‐born individuals with higher income and education more often believed they were likely to develop cancer and worry about getting cancer. Sixty eight percent of respondents were up‐to‐date with colorectal, 44% with breast, and 61% with cervical cancer screening. Multivariate models showed that higher education, lack of fatalism, older age, lower household income, and unmarried status were associated with cervical cancer screening adherence.

**Conclusions:**

Among a Hispanic population in the state of Indiana, factors associated with cervical cancer screening adherence were similar to the general population, with the exceptions of income and marital status. Younger Hispanic individuals were more likely to be adherent with breast and colorectal cancer screening, and given the higher incidence of cancer among older individuals, these results should guide future research and targeted outreach.

## Graphical abstract

Younger age was associated with higher likelihood of being up‐to‐date with breast and colorectal cancer screening while younger age, higher education, married status and lower income were associated with higher likelihood of being up‐to‐date with cervical cancer screening in Hispanics living in Indiana.

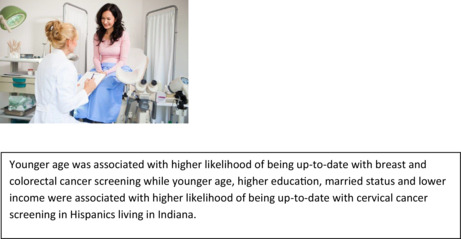

## 1 INTRODUCTION

According to the American Cancer Society, cancer is the leading cause of death for Hispanics in the United States and in Indiana, accounting for 21% and 19% of deaths in 2016, respectively.^1^ While Hispanics in Indiana have lower incidence and mortality rates from cancer compared to non‐Hispanic whites on a national level,^2^ cervical cancer incidence rates among Hispanics are 40% higher than those of non‐Hispanic whites, and liver and stomach cancer incidence rates are double.^1^ Additionally, Hispanics are more likely to be diagnosed with cancer at a later stage. From 2011 to 2015, 48% of Hispanic women with breast cancer were diagnosed at an early stage compared to 54% of non‐Hispanic white women. Hispanic women were also less likely to engage in timely follow‐up of abnormal mammography screenings and breast cancer treatment, possibly due to barriers in accessing healthcare.^2^

High cancer rates among Hispanics within Indiana and the United States may be attributed to various causes. First, Hispanics are less likely to have health insurance.^3^ According to the Indiana Behavioral Risk Factor Surveillance System (BRFSS), in 2016, 39% of Hispanics aged 18–64 were uninsured, compared to 10.8% of non‐Hispanic whites; this leads to lower screening rates among Hispanics in general and uninsured/underinsured Hispanics in particular which contributes to increased cancer rates in this population. Second, Hispanics are more likely to experience higher rates of poverty and limited transportation which can also decrease access to services. Nearly 30% of Hispanic residents in Indiana were living below the poverty line between 2010–2014 (compared to 13% of non‐Hispanic whites).^2^ Risky health behaviors, obesity, physical inactivity,^4^ and alcohol consumption^5^ have been noted to occur at higher rates among Hispanics, which contribute to high cancer incidence. According to the Centers for Disease Control (CDC), in 2020, 40% of Hispanics in Indiana self‐reported as obese, compared to 35.1% of non‐Hispanic whites.^6^

Cancer morbidity and mortality can be decreased by cancer screening, including mammograms,^7^ pap smears,^8^ and tests for colorectal cancer.^9^ At a national level, Hispanics have equivalent rates of breast (78.9% vs. 78.1%) and cervical (80.3% vs. 80%) cancer screening and lower rates of colorectal cancer screening (60.8% vs. 72.8%) when compared to non‐Hispanic whites.^10^ Hispanics in Indiana have participated in cancer screening to varying degrees. According to the National Cancer Institute, in 2016, only 62.2% of Hispanics (aged 50 or older) in Indiana reported that they were up‐to‐date with colorectal cancer screening (had fecal occult blood test in the last year, sigmoidoscopy in the last 5, or a colonoscopy in the last 10 years). In 2016, 75.1% of Hispanic women in Indiana (aged 40 or older) had received a mammogram in the previous 2 years, and only 66.9% of women (aged 18 or older) had received a pap smear in the previous 3 years.^11^ This stands in comparison with national averages, where in 2020 69.4% of US adults were up to date with colorectal cancer screening,^12^ in 2019 76.4% of women aged 50–74 years had a mammogram within the past 2 years^13^ and 73.5% of women aged 21–65 years were up to date with cervical cancer screening.^14^

In this study, we sought to describe more recent patterns of screening among the Indiana Hispanic population, as well as characterize what factors are associated with cancer screening (socioeconomic status, knowledge, and beliefs). We hypothesized that patients from more socially vulnerable populations (non‐US nationality, rural, lower income, and lower education) were less likely to have accurate cancer screening knowledge and adherence; additionally, we hypothesized that more accurate knowledge was associated with adherence.

In way of background, in 2018, our research team conducted a statewide survey to examine Indiana residents' knowledge, beliefs, and behaviors regarding cancer screening and prevention strategies. Results revealed socioeconomic and racial disparities in health behaviors and receipt of cancer screening,^15^ as well as differences in health information technology use between rural and urban populations.^16^ Data were collected from residents in 34 Indiana counties with higher cancer mortality rates than the state average, but this study did not reveal significant trends among Indiana Hispanics; in part due to under‐representation of this minority group, as Hispanic participation accounted for only 1.75% of respondents (unpublished data) while in 2014, Hispanics constituted 6.45% of the state's population, furthermore, recent data suggest they now represent 7.3% (approximately 495,343 people).^17^

Yet little is known about Hispanics' health and cancer‐related health behaviors; thus, more research is needed to understand the needs of this population. Bridging this knowledge gap will serve Indiana healthcare and public health organizations in better identifying the health needs of the Hispanic population. The objectives of this study were to describe cancer knowledge, beliefs (cancer risk perception and fatalism), and cancer screening behaviors among Hispanic adults residing in Indiana, and to examine differences by country of birth, age, marital status, cancer fatalism, socioeconomic status, and area of residence (urban vs. rural).

## 2 METHODS

A cross‐sectional survey was used to assess cancer‐related knowledge and beliefs, information‐seeking behaviors, and cancer screening among Hispanic adults in Indiana. Eligible participants were Hispanic adults (over age 18) living in the state of Indiana who could read and write either English or Spanish. Participants were recruited using Facebook‐targeted advertisement and surveys were available from August 12, 2021, until October 6, 2021. Data were collected through English and Spanish language surveys developed in Qualtrics. This survey had no informed consent and consent was implied from completion of the survey. Agreement to participate, after reading the conditions of participation and confidentiality, signified agreement to take part in a non‐identifiable role in a low‐risk instrument. Their continuation with the survey was taken as their consent. Upon survey completion, participants received a $15 electronic gift card. The Indiana University‐Purdue University Indianapolis IRB approved the study protocol (IUSCC‐2002388780). We leveraged Qualtrics IP‐detection technology to avoid duplicates and screen out potential respondents outside of our catchment area.

### 2.1 Measures

To develop the survey instrument, we refined a population health survey tool that had been used in 2017 on all ethnic/race groups in Indiana funded by the National Cancer Institute (P30 CA082709‐17S6). The 2017 survey examined knowledge, beliefs, and behaviors associated with cancer screening and cancer prevention strategies among 980 Indiana adults.^15^, ^16^, ^18^ Relevant survey items were identified from the Health Information National Trends Survey (HINTS), the Behavioral Risk Factor Surveillance System (BRFSS) survey, and the National Health and Nutrition Examination Survey (NHANES). Survey items assessed the following areas: (1) individual and sociodemographic characteristics, (2) cancer knowledge and beliefs; (3) cancer screening; and (4) health information‐seeking behaviors and preferences.^19^ Rural status was calculated using respondent zip codes and USDA (United States Department of Agriculture) defined RUCA (Rural–Urban Commuting Area) codes, then pairing them with census data.

Cancer fatalism is defined as deterministic beliefs about cancer, including the powerlessness of humans to influence cancer outcomes, the definitive role of external causes in the development of cancer, and the inevitability of death after a cancer diagnosis; we measure this by using four questions in our instrument, noted in Table 2 under the cancer fatalism category.^20^

For this study, we created a Hispanic/Spanish adaptation of the original 2017 survey instrument in both English and Spanish. Five bilingual members of the study team formed a Translation Task Force that translated, revised, and finalized the Spanish language survey and study documents (study information sheets, consent forms, etc.). Prior to recruitment, we conducted 10 cognitive interviews (five in Spanish and five in English). Cognitive interviews focused on the structure, flow, and layout of the survey, as well as the wording, order, and clarity of the questions and images; cognitive interviews are a well‐established method to refine questionnaires.^21^ Interviewees were selected from a convenience sample and included both English and Spanish‐speaking Hispanic adults residing in Indiana. Comments and suggestions made by interviewees were used to revise and finalize both versions of the survey (English and Spanish).

### 2.2 Recruitment

We developed our online recruitment approach after consulting with an experienced researcher who specializes in social media‐based research recruitment.^22^ First, we created a study page on Facebook. The page contained a description of the study, along with its aims and objectives. This page also contained contact information for study personnel and explained how interested members of the target population could participate in the study. Next, we used Facebook's marketing features to distribute two targeted ads—one in English and one in Spanish. The ads included a generic description of the study and a hyperlink to the eligibility screening questionnaire. Eligibility screening questions asked respondents their age, whether they currently resided in Indiana, and whether they identified as Hispanic, Latino, or of Spanish origin. Upon completing the screening questionnaire, eligible participants were directed to the study survey.

### 2.3 Data collection

Data collection took place during the COVID‐19 pandemic; we adapted our approach to the needs for social distancing. Both the Spanish and English language surveys included 78 closed‐ and 15 open‐ended items. The average time required to complete the survey was 57.4 minutes (SD = 88.6). We designed the survey so that eligible participants would take the survey only once through: the “Prevent Ballot Box Stuffing” option in Qualtrics. Prior to distributing compensation, we manually reviewed each participant's survey to verify its completion, and whether the zip code provided was in Indiana.

Two weeks after publishing our Facebook‐targeted advertisement, we evaluated the number of completed surveys in each language, and the age, gender, and zip code distributions of participants. We addressed language and age imbalances through pausing the English language ad and survey and continued invitations to target Spanish‐speaking participants aged 50 and older. In addition to releasing a revised Facebook advertisement, we also promoted the study on a local Spanish language television news broadcast to encourage Spanish‐speaking Hispanics in Indiana aged 50 and over to participate in the survey. Survey completion rates and limited demographic data were again reviewed 2 weeks later. After receiving 850 Spanish language and 791 English language surveys (1641 total), we closed both surveys. A total of 1086 individuals took the Spanish language survey, but we eliminated 236 that were incomplete. A total of 851 individuals completed English language surveys, but we eliminated 60 that were incomplete zip codes that were not in Indiana. Only surveys confirmed as “finished” by Qualtrics, and only those completed by Indiana residents who identified as either Latino, Hispanic, or Spanish were used. The total number of usable surveys was 1520 (93% of surveys collected).

### 2.4 Data analysis

To describe knowledge, beliefs, and behaviors regarding cancer screening, we calculated frequencies and percentages for all relevant questions. Screening guideline adherence, or being up‐to‐date with screening, for all three cancer types was calculated using the 2016 U.S. Preventive Services Task Force screening guidelines.^23^, ^24^, ^25^ We then compared knowledge, beliefs, and screening behaviors based on nationality, age, cancer fatalism, income, education, and area of residence (urban vs. rural) using Chi‐square or Fisher's exact tests depending on counts by categories. Logistic regression models were used for multivariate analysis. To identify factors associated with being adherent to screening guidelines, we performed univariate models to screen for potentially related variables (defined as those with a *p*‐value≤0.2). We then included factors found to be univariately significant in separate multivariate logistic regression models for each screening outcome. All statistical analyses were performed in R v4.0.3 (R Core Team, 2013) and RStudio v1.2.1335 (RStudio Team, 2015) using base packages and the car package.^26^

## 3 RESULTS

The final survey sample consisted of 1520 Hispanic respondents. The mean age of respondents was 47 (SD = 12.5) and the median age 53. Sex was evenly distributed across the population (52% completed by men and 48% by women). The proportion of respondents who chose to fill out the survey in English was 49.1%, while 50.9% completed in Spanish. The nationality (nation of birth) of respondents was as follows: USA (60.4%), Mexico (13.7%), Cuba (7.1%), Puerto Rico (5.7%), and other (13.2%). The distribution of income, educational levels, and other key sociodemographic characteristics are described in Table 1.

**TABLE 1:** Sample characteristics

Variable	Mean	Standard deviation	Median	*n*
Age (years)	47.26	12.52	53	1517
Sex	Frequency		Percentage	
Female	726		47.76	
Education				
Less than high school	153		10.07	
Completed high school	663		43.62	
Some college	573		37.70	
Completed college	131		8.62	
Rent or own home?				
Rent	534		35.13	
Own	684		45.00	
Occupy home with no rent	302		19.87	
Marital/partnered status				
Married or partnered	1332		87.63	
Not married or partnered	188		12.37	
Language spoken with partner				
All/mostly English	1157		80.91	
All/mostly Spanish	197		13.78	
Spanish/English equally	76		5.31	
Race				
White	1073		70.22	
Black	23		1.51	
Multiracial	91		5.96	
Indigenous or Mestizo	169		11.06	
Other	172		11.26	
Nationality				
Cuba	108		7.11	
Mexico	208		13.68	
Puerto Rico	86		5.66	
USA	918		60.39	
Other	200		13.16	
Annual household Income				
$0–34,999	125		8.26	
$35,000–49,999	548		36.22	
$50,000–74,999	635		41.97	
$75,000+	205		13.55	
Financial adequacy				
Finding it difficult/very difficult to get by on present income	717		47.20	
Getting by on present income	597		39.30	
Comfortable on present income	205		13.50	
How often did you not have enough money to buy food in the past 6 months?	Frequency		Percentage	
Rarely	948		62.41	
Never	235		15.47	
Other	336		22.12	
How often did you skip meals in the past 6 months?				
Rarely	954		62.80	
Never	276		18.17	
Other	289		19.03	
Employment status				
Employed full‐time	546		35.92	
Employed part‐time	617		40.59	
Unemployed	224		14.74	
Retired, disabled, or otherwise not working	133		8.75	
Area of residence				
Urban	1217		80.07	
Rural	303		19.93	

A minority of adults were able to correctly estimate the ages when cancer screening should begin, ranging from 4% who correctly identified age 55 to begin screening for lung cancer to 18% who identified the correct age to begin screening for breast cancer (Table 2). However, despite this inaccurate knowledge, they were more often adherent with cancer screening, ranging from 44% (lung cancer) to 69% (colorectal cancer, Table 2; Cancer Screening). Participants were unable to correctly identify cancer screening guidelines, regardless of what groups were considered (Tables 2, 3, 4). There were significant differences in cancer knowledge by education level, but almost no differences by nationality, urban/rural status, or income. Compared to participants without a high school education, a greater proportion of participants who completed college accurately identified the age to begin cancer screening for colon (30.4% vs. 17.9% *X* ^2^ = 19.2, df = 3, *p* < 0.001) and lung cancer (6.9% vs. 0.7% *X* ^2^ = 11.6, df = 3, *p* = 0.009), although participants with lower educational attainment were more often accurate about the age to begin breast cancer screening (30.8% vs. 11.1%, *X* ^2^ = 12.3, df = 3, *p* = 0.007).

**TABLE 2:** Cancer knowledge, beliefs, and screening behaviors among Hispanic adults in Indiana

Variable	Mean	SD	Median	Min	Max	*n*	Recommended age
At what age are most people supposed to start doing home blood stool tests, having a sigmoidoscopy or having a colonoscopy?	46.31	4.82	45	2	62	808	50
At what age should long‐term cigarette smokers start having a lung cancer screening test?	47.55	7.20	49	17	66	1235	55
At what age are most women supposed to start having mammograms?	45.95	5.26	45	20	60	439	40

	Frequency	Percentage
Cancer knowledge		
Correctly identified age to begin colon cancer screening	130	13.51
Correctly identified age to begin lung cancer screening (in long‐term smokers)	65	4.28
Correctly identified age to begin breast cancer screening	91	18.27

Cancer beliefs		
How likely are you to get cancer in your life?		
Likely	440	28.95
Neither likely nor unlikely	186	12.24
Unlikely	894	58.82
How worried are you about getting cancer?		
Extremely/moderately	512	33.68
Somewhat	552	36.32
Slightly/not at all	456	30.00
It is hard to know which cancer prevention recommendations to follow (Agree)	920	60.53
Cancer fatalism		
It seems like everything causes cancer (Agree)	838	55.13
When I think about cancer, I think about death (Agree)	857	56.38
You cannot lower your cancer risk much (Agree)	809	53.22
I did rather not know my cancer risk (Agree)	821	54.01
Up‐to‐date with cancer screening?		
Colorectal cancer: Yes	618	68.59
Breast cancer: Yes	185	44.15
Cervical cancer: Yes	407	61.20

Significant differences were observed in cancer beliefs when stratified as above (Tables 3 and 4; Beliefs). Income was significantly associated with participant beliefs; responses to every individual belief question were significantly different across income levels (Table 4). Additionally, significant differences were observed across nationalities and education levels, but few differences were observed between rural and urban areas of residence (Table 3).

**TABLE 3:** Cancer knowledge, beliefs, and screening behaviors by nationality and area of residence

	Nationality *n* (%)						Area of residence *n* (%)		
	USA	Mexico	Cuba	Puerto Rico	Other	*p*‐value	Urban	Rural	*p*‐value
Cancer knowledge									
Correctly identified age 50 to begin colon cancer screening	76 (15.8)	22 (13)	10 (10.2)	8 (10.8)	14 (10.1)	0.296	103 (13.7)	27 (12.8)	0.730
Correctly identified age 55 to begin lung cancer screening	46 (5.0)	2 (1.0)	5 (4.6)	2 (2.3)	10 (5.0)	0.054 *	52 (4.3)	13 (4.3)	0.989
Correctly identified age 40 to begin breast cancer screening	41 (16.8)	21 (24.7)	8 (17)	11 (26.2)	10 (12.5)	0.177	72 (18.4)	19 (17.9)	0.917
Cancer beliefs									
How likely are you to get cancer in your life?									
Likely	281 (30.6)	41 (19.7)	22 (20.4)	18 (20.9)	78 (39.0)	<0.001	365 (30)	75 (24.8)	0.113
Neither likely nor unlikely	108 (11.8)	24 (11.5)	7 (6.5)	5 (5.8)	42 (21.0)		152 (12.5)	34 (11.2)	
Unlikely	529 (57.6)	143 (68.8)	79 (73.1)	63 (73.3)	80 (40.0)		700 (57.5)	194 (64.0)	
How worried are you about getting cancer?									
Extremely/moderately	326 (35.5)	55 (26.4)	28 (25.9)	15 (17.4)	88 (44.0)	<0.001	434 (35.7)	78 (25.7)	0.004
Somewhat	377 (41.1)	65 (31.3)	32 (29.6)	27 (31.4)	51 (25.5)		432 (35.5)	120 (39.6)	
Slightly/not at all	215 (23.4)	88 (42.3)	48 (44.4)	44 (51.2)	61 (30.5)		351 (28.8)	105 (34.7)	
It is hard to know which cancer prevention recommendations to follow (Agree)	541 (58.9)	140 (67.3)	61 (56.5)	54 (62.8)	124 (62.0)	0.193	726 (59.7)	194 (64)	0.164
Cancer fatalism									
It seems like everything causes cancer (Agree)	548 (59.7)	99 (47.6)	49 (45.4)	47 (54.7)	95 (47.5)	<0.001	675 (55.5)	163 (53.8)	0.601
When I think about cancer, I think about death (Agree)	533 (58.1)	114 (54.8)	50 (46.3)	49 (57.0)	111 (55.5)	0.214	695 (57.1)	162 (53.5)	0.253
You cannot lower your cancer risk much (Agree)	502 (54.7)	108 (51.9)	61 (56.5)	43 (50.0)	95 (47.5)	0.357	644 (52.9)	165 (54.5)	0.631
I would rather not know my cancer risk (Agree)	517 (56.3)	103 (49.5)	54 (50.0)	46 (53.5)	101 (50.5)	0.252	652 (53.6)	169 (55.8)	0.492
Up‐to‐date with cancer screening?									
Colorectal cancer: Yes	298 (68.3)	116 (70.7)	62 (63.9)	53 (74.6)	89 (66.9)	0.605	496 (70)	122 (63.5)	0.089
Breast cancer: Yes	94 (50.0)	34 (45.3)	18 (40.9)	17 (43.6)	22 (30.1)	0.070	146 (44.4)	39 (43.3)	0.860
Cervical cancer: Yes	256 (65.6)	56 (57.1)	22 (48.9)	25 (58.1)	48 (53.9)	0.063	330 (61.8)	77 (58.8)	0.525

*Note*: *p*‐values were calculated from Chi‐square test, except those denoted with an asterisk (*) that were calculated from Fisher's exact test.

**TABLE 4:** Cancer knowledge, beliefs, and screening behaviors by income and education

	Income *n* (%)					Education *n* (%)				
	0 to $34,999	$35,000 to $49,999	$50,000 to $74,999	$75,000+	*p*‐value	Less than high school	Completed high school	Some college or university	Completed college or higher	*p*‐value
Cancer knowledge										
Correctly identified age 50 to begin colorectal cancer screening	12 (13.3)	68 (20.9)	37 (9.5)	12 (7.7)	<0.001	24 (17.9)	56 (10.2)	36 (15.3)	14 (30.4)	<0.001
Correctly identified age 55 to begin lung cancer screening	6 (4.8)	18 (3.3)	31 (4.9)	10 (4.9)	0.544	1 (0.7)	22 (3.3)	33 (5.8)	9 (6.9)	0.005 *
Correctly identified age 40 to begin breast cancer screening	13 (24.5)	40 (23.0)	30 (15.2)	8 (11.6)	0.064	24 (30.8)	47 (18.4)	16 (12.5)	4 (11.1)	0.009 *
**Cancer beliefs**										
How likely are you to get cancer in your life?										
Likely	26 (20.8)	103 (18.8)	230 (36.2)	79 (38.5)	<0.001	37 (24.2)	162 (24.4)	183 (31.9)	58 (44.3)	<0.001
Neither likely nor unlikely	18 (14.4)	62 (11.3)	73 (11.5)	29 (14.1)		12 (7.8)	52 (7.8)	79 (13.8)	43 (32.8)	
Unlikely	81 (64.8)	383 (69.9)	332 (52.3)	97 (47.3)		104 (68.0)	449 (67.7)	311 (54.3)	30 (22.9)	
It is hard to know which cancer prevention recommendations to follow (Agree)	82 (65.6)	302 (55.1)	399 (62.8)	131 (63.9)	0.015	94 (61.4)	398 (60.0)	337 (58.8)	91 (69.5)	0.157
**How worried are you about getting cancer?**										
Extremely/moderately	29 (23.2)	152 (27.7)	252 (39.7)	78 (38.0)	<0.001	36 (23.5)	201 (30.3)	220 (38.4)	55 (42.0)	<0.001
Somewhat	54 (43.2)	239 (43.6)	203 (32.0)	55 (26.8)		44 (28.8)	228 (34.4)	244 (42.6)	36 (27.5)	
Slightly/not at all	42 (33.6)	157 (28.6)	180 (28.3)	72 (35.1)		73 (47.7)	234 (35.3)	109 (19.0)	40 (30.5)	
**Cancer fatalism**										
It seems like everything causes cancer (Agree)	74 (59.2)	318 (58.0)	347 (54.6)	95 (46.3)	0.027	87 (56.9)	328 (49.5)	344 (60.0)	79 (60.3)	0.001
When I think about cancer, I think about death (Agree)	82 (65.6)	332 (60.6)	341 (53.7)	97 (47.3)	0.001	83 (54.2)	358 (54.0)	345 (60.2)	71 (54.2)	0.140
You cannot lower your cancer risk much (Agree)	49 (39.2)	278 (50.7)	366 (57.6)	113 (55.1)	0.001	87 (56.9)	352 (53.1)	309 (53.9)	61 (46.6)	0.353
I would rather not know my cancer risk (Agree)	67 (53.6)	319 (58.2)	344 (54.2)	87 (42.4)	0.002	73 (47.7)	356 (53.7)	339 (59.2)	53 (40.5)	<0.001
**Up‐to‐date with cancer screening?**										
Colorectal cancer: Yes	67 (77.0)	211 (71.5)	244 (66.1)	95 (63.8)	0.082	87 (65.9)	363 (69.9)	142 (67.9)	26 (63.4)	0.698
Breast cancer: Yes	28 (60.9)	70 (51.1)	68 (39.5)	19 (30.2)	0.002	36 (48.6)	89 (38.9)	49 (52.7)	11 (47.8)	0.108
Cervical cancer: Yes	47 (75.8)	175 (70.3)	138 (52.3)	44 (52.4)	<0.001	40 (52.6)	156 (57.4)	166 (66.4)	45 (67.2)	0.048

*Note*: *p*‐values were calculated from Chi‐square test, except those denoted with an asterisk (*) that were calculated from Fisher's exact test.

When examining adherence to cancer screening guidelines, that is, report of being up‐to‐date with screening, there was no association between higher educational attainment and screening adherence, except in the case of cervical cancer: (*X* ^2^ = 7.9, *p* = 0.048). Income level was significantly associated with reported screening adherence, whereby individuals with lower incomes were reported being more adherent with breast (*X* ^2^ = 14.4, *p* = 0.002) and cervical (*X* ^2^ = 25.9, *p* < 0.001) cancer screening guidelines (Table 4).

With regard to other factors associated with reported cancer screening adherence, younger age (OR = 0.96, 95%CI [0.92–1.00], *p* = 0.031) and urban residency (OR = 0.68, 95%CI [0.48–0.97], *p* = 0.032) were associated with higher odds of being adherent to colorectal cancer screening, while primarily Spanish language usage was found to be associated with lower odds of adherence. Younger age (OR = 0.93, 95%CI [0.88–0.99], *p* = 0.019) and white race (OR = 2.25, 95%CI [1.32–3.90] *p* = 0.003) were associated with higher odds of being adherent to breast cancer screening. Finally, women who disagree that they think of cancer as fatal (OR = 0.64, 95%CI [0.45–0.91] *p* = 0.012), non‐married women (OR = 0.57, 95%CI [0.33–0.96], *p* = 0.035), and those with higher incomes (OR = 0.38, 95%CI [0.17–0.82], *p* = 0.004) had lower odds of being adherent to cervical cancer screening. Older women (OR = 1.03, 95%CI [1.01–1.04], *p* = 0.008), and those that completed college or higher education (OR = 4.53, 95%CI [2.02–10.43], *p* < 0.001) had higher odds of being adherent to cervical cancer screening guidelines (Table 5).

**TABLE 5:** Multivariate, logistic regression models predicting cancer screening adherence

Colorectal cancer screening (*n* = 892)	Odds ratio (95% CI)	*p*‐value
Age	**0.96 (0.92–1.00)**	**0.031**
Language spoken with partner		**0.029**
All or mostly English (*n* = 827)	(ref)	
All or mostly Spanish (*n* = 42)	**0.45 (0.23–0.89)**	
Spanish and English equally (*n* = 23)	1.79 (0.70–5.06)	
Area of residence		**0.032**
Urban (*n* = 703)	(ref)	
Rural (*n* = 189)	**0.68 (0.48–0.97)**	
Breast cancer screening (*n* = 413)		
Age	**0.93 (0.88–0.99)**	**0.019**
Race		**0.003**
Non‐white (*n* = 126)	(ref)	
White (*n* = 287)	**2.25 (1.32–3.90)**	
Cervical cancer screening (*n* = 659)		
Age	**1.03 (1.01–1.04)**	**0.008**
When I think about cancer, I think about death		**0.012**
Agree (*n* = 371)	(ref)	
Disagree (*n* = 288)	**0.64 (0.45–0.91)**	
How likely are you to get cancer in your life?		**0.023**
Likely (*n* = 184)	(ref)	
Neither likely nor unlikely (*n* = 83)	1.42 (0.80–2.54)	
Unlikely (*n* = 392)	**1.82 (1.19–2.79)**	
Education		**<0.001**
Less than high school (*n* = 75)	(ref)	
Completed high school (*n* = 271)	1.53 (0.87–2.68)	
Some college (*n* = 249)	**2.71 (1.43–5.19)**	
Completed college (*n* = 64)	**4.53 (2.02–10.43)**	
Annual household income		**0.004**
$0–34,999 (*n* = 62)	(ref)	
$35,000–49,999 (*n* = 249)	0.56 (0.27–1.13)	
$50,000–74,999 (*n* = 264)	**0.34 (0.16–0.67)**	
$75,000+ (*n* = 84)	**0.38 (0.17–0.82)**	
Marital/partnered status		**0.035**
Married/partnered (*n* = 581)	(ref)	
Not married/partnered (*n* = 78)	**0.57 (0.33–0.96)**	

*Note*: Only showing significant results. The three multivariate models included the following variables: Colorectal cancer (Age, “Cancer worries me (y/n),” “How likely are you to get cancer in your life?,” “Everything seems to cause cancer,” financial adequacy, annual household income, correctly identified age to begin lung cancer screening, language spoken with partner, marital status, “How often did you not have enough money to buy food in the past 6 months?,” Race, “How often did you skip meals in the past 6 months?,” and area of residence). Breast cancer (age, “Cancer worries me (y/n),” “How likely are you to get cancer in your life?,” education level, annual household income, language spoken with partner, nationality, race, rent or own home, and area of residence). Cervical cancer (age, “When I think about cancer, I think about death (y/n),” “Cancer worries me (y/n),” “How likely are you to get cancer in your life?,” education level, “I've had the HPV vaccine (y/n),” annual household income, marital status, nationality, race, rent or own home, and area of residence). *p* ‐ Values and significant ORs providing 95% CI are in bold.

## 4 DISCUSSION

In this state‐based population study, we identified intra‐ethnic differences among the Hispanic population in Indiana that had not been previously described. This finding can be understood through the lens of both intersectionality and minority poverty hypotheses. The former states that multiple dimensions of social status interact and condition responses to risk factors and resources relevant to disease^27^ while the latter states that disparities are concentrated in minority groups with low socioeconomic status (SES).^28^ Using this framework, our results characterize the intra‐ethnic difference found as a function of educational level and SES.

Among the Hispanic population in Indiana, Hispanics originally from Mexico constitute 4.9% of the Hispanic population, 0.1% were from Cuba, and the remaining 1.65% were described as other Hispanic or Latinos.^29^ Other substantive disparities for age and educational level are also apparent.^30^ Hispanics in the United States have high rates of chronic health conditions such as obesity,^31^ hypertension,^32^ diabetes,^33^ liver disease,^34^ kidney disease,^35^ and cancer,^36^ which resemble the larger health issues for Hispanics in Indiana.

This study's Hispanic population frequently did not accurately identify the appropriate ages to start screening for lung, breast, and colorectal cancer. On average, individuals answered that cancer screening should begin at a younger age than USPSTF guidelines recommend. This discrepancy could be secondary to the lack of dissemination of appropriate information about screening guidelines within the Hispanic community^37^ as well as changes in screening guidelines,^38^, ^39^ particularly in regards to colon cancer, where both the USPSTF^40^ and the American College of Gastroenterology^41^ have recently recommended to initiate screening at younger ages for adults at average risk which in turn could further complicate dissemination of updated information. Prior study of Hispanic perceptions about CRC (colorectal cancer) screening suggest the need for messages in both Spanish and English that illustrate and define key terms (e.g., polyp), include basic information about cancer screening as well as available screening options, address individual risk perception, and use familial images.^42^ Participants with lower educational attainment were more likely to be inaccurate, except for breast cancer screening, which has been noted in different settings, likely as a result of higher promotion and awareness of breast cancer screening.^43^ Additionally, the deployment of targeted social support interventions has been shown to increase knowledge and adherence with cancer screening,^44^ which provides a possible avenue to remedy these disparities. While educational level was positively associated with knowledge, it did not correlate with reported adherence to screening guidelines, with the exception of cervical cancer; this unexpected lack of correlation between knowledge and reported adherence has also been noted with regard to colon cancer screening in black men and hypothesized to be due to fear of potential results or perhaps due to individuals being action‐oriented problem solvers rather than prevention‐oriented.^45^

Paradoxically, respondents with lower incomes were more likely to be adherent to screening guidelines than those with higher income. This result differs from other studies which found that higher income was associated with higher rates of cancer screening^46^, ^47^ and lower income with lower rates of screening.^48^ However, none of these studies looked exclusively at the Hispanic population, and definitions of lower income differed between studies. There is also a possibility that social desirability bias (making individuals more likely to report screening if perceived as the appropriate action) may operate differentially among low‐ and high‐income groups; additionally, it is possible that acculturation, which was not measured by this survey, could play a part in these outcomes, as Hispanic people with higher acculturation levels have in general higher incomes,^49^ and have been noted to have worse self‐reported physical health^50^ and lower rates of healthy behavior^51^ when compared to less acculturated Hispanics, however, this inverse impact of acculturation on cancer screening has been inconsistent.^52^ Another important negative finding was the lack of significant differences in reported cancer screening adherence among urban and rural Hispanic populations. Prior studies suggest that cancer screening is lower among rural populations in general,^53^, ^54^ but again, rurality was not a significant determinant among this Hispanic population, perhaps due to the acculturation effect noted above.

Higher income and education groups, as well as US‐born individuals, more often believed that they were likely to develop cancer and worried about getting cancer. These elevated cancer risk perceptions, especially among respondents with higher socioeconomic status, do not align with epidemiologic reality, wherein there is a moderately strong, inverse association between income level and cancer incidence.^55^, ^56^ Higher socioeconomic status has previously been associated with greater perceptions of lung cancer risk^57^ and the harms of smoking,^58^ and black race with lower perceptions of the risk of developing breast cancer.^59^ Our study is unique in assessing cancer beliefs among Hispanics, and we found a similar, direct association between higher socioeconomic status and greater risk perception. Notably, these perceptions are not necessarily accurate, as a substantial proportion of individuals at average risk for cancer perceive themselves at increased risk,^60^ in other disease contexts, this phenomenon is sometimes referred to as the “worried well”.^61^

With regard to cancer fatalism, lower income individuals were more likely to agree with the statements: “When I think about cancer, I think about death” and “You cannot lower your cancer risk much”. Both lower income and lower education groups more often agreed that “I would rather not know my cancer risk”. Previous studies have found that cancer fatalism tends to be more prevalent among lower socioeconomic groups.^62^, ^63^ The twin beliefs of cancer fatalism, taken together with risk minimization, run the danger of reducing the uptake of beneficial cancer screening and prevention practices among low‐income populations.^20^ Public health practitioners should tailor messages to take these beliefs into account, as well as target high‐risk groups among whom cancer fatalism or risk minimization may serve as barriers to the uptake of evidence‐based cancer screening.

Our multivariate analysis showed that younger patients were more likely to adhere to breast and colorectal cancer screening guidelines when compared to older patients which may be due to lower health literacy among older Hispanics. Additionally, marital status, higher education level, and lower income also were associated with increased adherence to cervical cancer screening. Interestingly, the higher cervical cancer screening rates among low‐income respondents was an unexpected result; this could be a reflection of recent trends seeing increases in cancer screening rates among people with low socioeconomic status and the uninsured as a result of the Medicaid expansion under the Affordable Care Act, which Indiana implemented in 2015^64^; furthermore, the association between income level and cervical cancer screening rates has not been significant for Hispanic women—particularly those living in the Midwest—as compared to non‐Hispanic white women in prior studies.^65^ Differences in knowledge, beliefs, and behaviors in the Hispanic population highlight the need to better understand the relationships between sociodemographics and health behaviors among this population.

### 4.1 Limitations

The limitations of this study include its cross‐sectional design which prevents us from establishing causality between the health beliefs and knowledge measured and cancer screening adherence. The opt‐in nature of our recruitment introduces selection bias, as well as self‐report, social desirability and recall bias inherent to surveys, and the online sample does not necessarily represent a population‐based cohort of Hispanics in Indiana. However, we expect that our large sample size can at least partially counterbalance this through the improvement of statistical power in our analyses. Additionally, while our survey instrument was based on prior work, adopting items from other public surveys (e.g., HINTS, BRFSS, and NHANES), these items are lacking in external validation among Hispanic groups. While our study team applied robust translation practices and performed cognitive interviews among both English‐ and Spanish‐speaking Hispanic individuals, there is room for additional validation of these widely used instruments in the future. We also omitted certain questions for brevity and forecasted response rates, for example, knowledge assessment for age to start cervical and lung cancer screening were not included based on a predicted small amount of women age 21–65 respondents and on the relatively complex eligibility criteria (pack/year, time since cessation) for low‐dose CT (Computed Tomography), respectively. Finally, Hispanics residing in the Midwest are not necessarily representative of Hispanics residing in other areas of the United States due in part to variation in immigration patterns.

## 5 IMPLICATIONS/CONCLUSIONS

This study provides actionable information about cancer knowledge, beliefs, behaviors, and sociodemographic characteristics, based upon which cancer screening messages and interventions can be tailored to the most vulnerable sub‐groups. There appears a lack of knowledge regarding appropriate cancer screening guidelines among Hispanics in Indiana. The finding of higher rates of reported cancer screening adherence among lower income Hispanics is at odds with prior studies and merits further exploration and study. In general, these findings should be used to further emphasize the need of targeted educational interventions within this population as well as understanding the underlying beliefs of fatalism, risk minimization, and health literacy that will impact these potential interventions on at‐risk populations.

## AUTHOR CONTRIBUTIONS

**Manuel Ricardo Espinoza Gutarra:** Conceptualization (equal); formal analysis (equal); investigation (equal); methodology (equal); supervision (equal); validation (equal); writing – original draft (lead); writing – review and editing (lead). **Susan M Rawl:** Conceptualization (lead); data curation (equal); funding acquisition (equal); investigation (equal); methodology (lead); supervision (equal); visualization (equal); writing – original draft (equal); writing – review and editing (equal). **Gerardo Maupome:** Conceptualization (equal); data curation (equal); formal analysis (equal); funding acquisition (equal); investigation (equal); methodology (equal); supervision (equal); validation (equal); writing – review and editing (equal). **Heather O'Leary:** Conceptualization (equal); methodology (equal); supervision (equal); writing – review and editing (equal). **Robin E Valenzuela:** Conceptualization (equal); data curation (equal); formal analysis (equal); methodology (lead); project administration (lead); writing – review and editing (equal). **Caeli Malloy:** Conceptualization (equal); data curation (equal); formal analysis (equal); writing – review and editing (equal). **Lilian Golzarri‐Arroyo:** Data curation (equal); formal analysis (lead); software (equal); validation (equal). **Erik Parker:** Data curation (equal); formal analysis (lead); software (equal); validation (equal). **Laura Haunert:** Conceptualization (equal); data curation (equal). **David A Haggstrom:** Conceptualization (lead); data curation (equal); funding acquisition (equal); investigation (equal); methodology (equal); supervision (equal); visualization (equal); writing ‐ original draft (equal); writing ‐ review and editing (equal).

## FUNDING INFORMATION

This study was funded by the Indiana University Comprehensive Cancer Center. Caeli Malloy was supported by the National Institute of Nursing Research of the National Institutes of Health under Award No. T32NR018407. The content is solely the responsibility of the authors and does not necessarily represent the official views of the National Institutes of Health.

## CONFLICT OF INTEREST

The authors report no relevant conflict to interest.

## Supporting information


Table S1–S3

## Data Availability

The data that support the findings of this study are available on request from the corresponding author. The data are not publicly available due to privacy or ethical restrictions.
