# Supplementary material for: Cancer‐related knowledge, beliefs, and behaviors among Hispanic/Latino residents of Indiana
Source: Cancer Med. 2023 Jan 22;12(6):7470–84. doi: 10.1002/cam4.5466 (PMC10067073; doi:10.1002/cam4.5466)
Supplement: Supplementary file 1 — Table S1–S3 [file CAM4-12-7470-s001.docx]

Supplemental tables

Table S1. Full multivariate, logistic regression model predicting breast cancer screening adherence

|  | **Odds ratio (95% CI)** | **p-value** |
| --- | --- | --- |
| Age | **0.93 (0.88 - 0.99)** | **0.020** |
| How worried are you about getting cancer? |  | 0.129 |
| Extremely/Moderately (n = 112) | (ref) |  |
| Somewhat (n = 143) | 0.68 (0.38 - 1.21) |  |
| Slightly/Not at all (n = 158) | 1.11 (0.63 - 1.98) |  |
| How likely are you to get cancer in your life? |  | 0.8 |
| Likely (n = 103) | (ref) |  |
| Neither likely nor unlikely (n = 39) | 1.33 (0.57 - 3.06) |  |
| Unlikely (n = 271) | 1.10 (0.63 - 1.92) |  |
| Education |  | 0.351 |
| Less than high school (n = 74) | (ref) |  |
| Completed high school (n = 228) | 0.77 (0.43 - 1.36) |  |
| Some college (n = 91) | 1.25 (0.62 - 2.53) |  |
| Completed college (n = 20) | 1.07 (0.32 - 3.43) |  |
| Annual Household Income |  | 0.12 |
| $0 – 34,999 (n = 43) | (ref) |  |
| $35,000 – 49,999 (n = 137) | 0.65 (0.30 - 1.39) |  |
| $50,000 – 74,999 (n = 171) | 0.54 (0.25 - 1.18) |  |
| $75,000+ (n = 62) | **0.34 (0.13 - 0.85)** |  |
| Language spoken with partner |  | 0.149 |
| All or Mostly English (n = 374) | (ref) |  |
| All or mostly Spanish (n = 27) | 1.28 (0.50 - 3.22) |  |
| Spanish and English equally (n = 12) | 3.77 (0.98 - 17.19) |  |
| Nationality |  | 0.801 |
| USA (n = 184) | (ref) |  |
| Mexico (n = 74) | 1.06 (0.58 - 1.95) |  |
| Cuba (n = 43) | 1.00 (0.47 - 2.12) |  |
| Puerto Rico (n = 39) | 0.97 (0.45 - 2.05) |  |
| Other (n = 73) | 0.68 (0.35 - 1.32) |  |
| Race |  | **0.003** |
| Non-white (n = 126) | (ref) |  |
| White (n = 287) | **2.25 (1.32 - 3.90)** |  |
| Rent or own home? |  | 0.275 |
| Occupied without paying monetary rent (n = 135) | (ref) |  |
| Own (n = 153) | 0.82 (0.49 - 1.38) |  |
| Rent (n = 125) | 1.26 (0.73 - 2.17) |  |
| Area of residence |  | 0.875 |
| Urban (n = 326) | (ref) |  |
| Rural (n = 87) | 0.96 (0.57 - 1.61) |  |

Table S2. Full multivariate, logistic regression model predicting colorectal cancer screening adherence

|  | **Odds ratio (95% CI)** | **p-value** |
| --- | --- | --- |
| Age | **0.96 (0.92 - 1.00)** | **0.031** |
| How worried are you about getting cancer? |  | 0.539 |
| Extremely/Moderately (n = 258) | (ref) |  |
| Somewhat (n = 287) | 1.21 (0.82 - 1.78) |  |
| Slightly/Not at all (n = 347) | 1.22 (0.83 - 1.80) |  |
| How likely are you to get cancer in your life? |  | 0.08 |
| Likely (n = 208) | (ref) |  |
| Neither likely nor unlikely (n = 74) | 1.49 (0.83 - 2.72) |  |
| Unlikely (n = 610) | **1.55 (1.04 - 2.29)** |  |
| It seems like everything causes cancer |  | 0.73 |
| Agree (n = 429) | (ref) |  |
| Disagree (n = 463) | 1.06 (0.77 - 1.44) |  |
| Financial adequacy |  | 0.218 |
| Finding it difficult/very difficult to get by on present income (n = 539) | (ref) |  |
| Getting by on present income (n = 282) | 0.74 (0.53 - 1.05) |  |
| Comfortable on present income (n = 71) | 0.73 (0.39 - 1.36) |  |
| Correctly identified age to begin lung cancer screening |  | 0.55 |
| No (n = 864) | (ref) |  |
| Yes (n = 28) | 0.78 (0.35 - 1.78) |  |
| Annual Household Income |  | 0.279 |
| $0 – 34,999 (n = 83) | (ref) |  |
| $35,000 – 49,999 (n = 295) | 0.66 (0.35 - 1.19) |  |
| $50,000 – 74,999 (n = 367) | 0.57 (0.30 - 1.02) |  |
| $75,000+ (n = 147) | 0.57 (0.29 - 1.10) |  |
| Language spoken with partner |  | **0.029** |
| All or Mostly English (n = 374) | (ref) |  |
| All or mostly Spanish (n = 27) | **0.45 (0.23 - 0.89)** |  |
| Spanish and English equally (n = 12) | 1.79 (0.70 - 5.06) |  |
| Marital status |  | 0.526 |
| Married/partnered (n = 840) | (ref) |  |
| Not married/partnered (n = 52) | 0.82 (0.44 - 1.54) |  |
| How often did you not have enough money to buy food in the past 6 months? | 1.00 (0.47 - 2.12) | 0.305 |
| Never (n = 85) | (ref) |  |
| Other (n = 234) | 1.57 (0.85 - 2.89) |  |
| Rarely (n = 573) | 1.53 (0.88 - 2.65) |  |
| Race |  | 0.233 |
| Non-white (n = 262) | (ref) |  |
| White (n = 630) | 1.23 (0.88 - 1.72) |  |
| How often did you skip meals in the past 6 months? |  | 0.916 |
| Never (n = 105) | (ref) |  |
| Other (n = 200) | 1.11 (0.63 - 1.95) |  |
| Rarely(n = 587) | 1.03 (0.63 - 1.68) |  |
| Area of residence |  | **0.032** |
| Urban (n = 703) | (ref) |  |
| Rural (n = 189) | **0.68 (0.48 - 0.97)** |  |

Table S3. Full multivariate, logistic regression model predicting cervical cancer screening adherence

|  | **Odds ratio (95% CI)** | **p-value** |
| --- | --- | --- |
| Age | **1.03 (1.01 - 1.04)** | **0.008** |
| When I think about cancer, I think about death |  | **0.012** |
| Agree (n = 371) | (ref) |  |
| Disagree (n = 288) | **0.64 (0.45 - 0.91)** |  |
| How worried are you about getting cancer? |  | 0.202 |
| Extremely/Moderately (n = 205) | (ref) |  |
| Somewhat (n = 257) | 1.47 (0.95 - 2.26) |  |
| Slightly/Not at all (n = 197) | 1.34 (0.84 - 2.15) |  |
| How likely are you to get cancer in your life? |  | **0.023** |
| Likely (n = 184) | (ref) |  |
| Neither likely nor unlikely (n = 83) | 1.42 (0.80 – 2.54) |  |
| Unlikely (n = 392) | **1.82 (1.19 – 2.79)** |  |
| Education |  | **< 0.001** |
| Less than high school (n = 75) | (ref) |  |
| Completed high school (n = 271) | 1.53 (0.87 – 2.68) |  |
| Some college (n = 249) | **2.71 (1.43 – 5.19)** |  |
| Completed college (n = 64) | **4.53 (2.02 – 10.43)** |  |
| Had hpv vac |  | 0.111 |
| No (n = 356) | (ref) |  |
| Yes (n = 261) | **1.46 (1.01 - 2.12)** |  |
| Doctor refused when asked (n = 42) | 0.96 (0.46 - 1.97) |  |
| Annual Household Income |  | **0.004** |
| $0 – 34,999 (n = 62) | (ref) |  |
| $35,000 – 49,999 (n = 249) | 0.56 (0.27 – 1.13) |  |
| $50,000 – 74,999 (n = 264) | **0.34 (0.16 – 0.67)** |  |
| $75,000+ (n = 84) | **0.38 (0.17 – 0.82)** |  |
| Marital/Partnered status |  | **0.035** |
| Married/Partnered (n = 581) | (ref) |  |
| Not married/Partnered (n = 78) | **0.57 (0.33 – 0.96)** |  |
| Nationality |  | 0.201 |
| USA (n = 388) | (ref) |  |
| Mexico (n = 96) | 0.62 (0.37 - 1.06) |  |
| Cuba (n = 45) | 0.54 (0.27 - 1.09) |  |
| Puerto Rico (n = 43) | 0.57 (0.28 - 1.19) |  |
| Other (n = 87) | 0.85 (0.49 - 1.48) |  |
| Race |  | 0.064 |
| Non-white (n = 192) | (ref) |  |
| White (n = 467) | 1.47 (0.98 - 2.20) |  |
| Rent or own home? |  | 0.278 |
| Occupied without paying monetary rent (n = 181) | (ref) |  |
| Own (n = 256) | 0.72 (0.47 - 1.10) |  |
| Rent (n = 222) | 0.91 (0.58 - 1.44) |  |
| Area of residence |  | 0.233 |
| Urban (n = 529) | (ref) |  |
| Rural (n = 130) | 0.77 (0.50 - 1.18) |  |
